# Risk Factors for Treatment Failure of Drug-Susceptible Pulmonary Tuberculosis in Lithuania over 22 Years

**DOI:** 10.3390/medicina61101805

**Published:** 2025-10-08

**Authors:** Karolina Kėvelaitienė, Roma Puronaitė, Valerija Edita Davidavičienė, Birutė Nakčerienė, Edvardas Danila

**Affiliations:** 1 Institute of Clinical Medicine, Clinic of Chest Diseases, Immunology and Allergology, Vilnius University Faculty of Medicine, LT-03101 Vilnius, Lithuania; karolina.kevelaitiene@mf.vu.lt; 2 Centre of Pulmonology and Allergology, Vilnius University Hospital Santaros Klinikos, LT-08661 Vilnius, Lithuania; 3 Center of Informatics and Development, Vilnius University Hospital Santaros Klinikos, LT-08406 Vilnius, Lithuania; roma.puronaite@santa.lt; 4 Programs and Tuberculosis State Information System Department, Vilnius University Hospital Santaros Klinikos, LT-08661 Vilnius, Lithuania; edita.davidaviciene@santa.lt (V.E.D.); birute.nakceriene@santa.lt (B.N.)

**Keywords:** drug-susceptible tuberculosis management, tuberculosis risk factors, drug-susceptible tuberculosis treatment outcome

## Abstract

*Background and Objectives*: This study aimed to evaluate the treatment outcomes of adults with pulmonary drug-susceptible tuberculosis (DS-TB) in Lithuania over 22 years, and to examine associations between treatment outcomes, various risk factors, and temporal trends. *Materials and Methods*: A retrospective cohort analysis was conducted using data from the National Tuberculosis Information System from 2000 to 2021. A total of 18,697 adult patients with DS-TB were included. Patients were grouped into three time periods: Period I (2000–2007), Period II (2008–2015), and Period III (2016–2021). Treatment outcomes were categorized as successful (treatment completed with recovery) or unsuccessful (patients who encountered treatment failure, died during treatment, or converted to drug-resistant tuberculosis). Associations with individual risk factors, including smoking, alcohol use, comorbidities, and sociodemographic variables, were analyzed. *Results*: Treatment success rates improved steadily across the study periods: 82.3% in Period I, 84.4% in Period II, and 87.6% in Period III. Mortality rates declined over time but remained substantial: 17.1%, 15.2%, and 12.0% in Periods I, II, and III, respectively. Non-lethal treatment failures decreased slightly (0.6%, 0.4%, and 0.4%). Multivariate analysis identified significant associations between treatment failure and multiple risk factors, including low BMI, male gender, unemployment, homelessness, smoking, alcohol and substance use, and comorbid conditions such as cancer, cardiovascular disease, chronic lung disease, diabetes mellitus, HIV, and renal failure. *Conclusions*: Treatment outcomes for DS-TB in Lithuania have improved over the past two decades; however, certain modifiable risk factors—such as low BMI, homelessness, substance use, and comorbidities—remain strongly linked to treatment failure. To further improve outcomes, targeted interventions such as nutritional support, housing programs, and integrated addiction services should be prioritized for high-risk groups within national TB control efforts.

## 1. Introduction

Tuberculosis (TB) remains a major global health threat, responsible for immense human and economic burden despite decades of focused control efforts [1,2]. The World Health Organization (WHO) provides standardized definitions and reporting guidelines to improve the classification and monitoring of TB cases and outcomes. “Drug-susceptible TB (DS-TB)” is defined as a bacteriologically confirmed or clinically diagnosed case of TB without evidence of resistance to rifampicin and isoniazid. In contrast, “drug-resistant TB (DR-TB)” refers to infections caused by Mycobacterium tuberculosis strains resistant to one or more anti-TB drugs. DR-TB includes several subtypes: “rifampicin-resistant TB (RR-TB)”, characterized by resistance to rifampicin detected by phenotypic or genotypic methods; “multidrug-resistant TB (MDR-TB)”, which is resistant to at least both rifampicin and isoniazid; and “extensively drug-resistant TB (XDR-TB)”, defined as MDR-TB with additional resistance to any fluoroquinolone and at least one second-line injectable agent (amikacin, kanamycin, or capreomycin) [3].

Treatment outcomes are also clearly defined by the WHO. For DS-TB, “treatment failure” refers to a patient whose sputum smear or culture remains positive at month five or later during treatment. A “successful treatment” outcome includes patients who are cured or who completed treatment, regardless of whether bacteriological confirmation of cure was obtained at the end of therapy. Conversely, “unsuccessful treatment” outcomes include cases of treatment failure, death, loss to follow-up, or those not evaluated [3].

In 2023, TB once again became the leading cause of death from a single infectious agent, surpassing HIV/AIDS and marking a sobering return after a temporary decline during the COVID-19 pandemic [4]. Globally, an estimated 8.2 million new TB cases were reported in 2023, up from 7.5 million in 2022, continuing an upward trend observed since 2021 [5]. While much attention has rightfully been directed toward drug-resistant tuberculosis (DR-TB), a significant proportion of treatment failures still occur among patients infected with drug-susceptible tuberculosis (DS-TB). These cases often receive less scrutiny but carry substantial public health implications. Treatment failure in DS-TB prolongs infectiousness, worsens patient outcomes, increases healthcare costs, and contributes to the development and spread of drug resistance. Moreover, failed treatment in DS-TB patients, who are generally expected to respond well to first-line therapy, may serve as an early indicator of systemic weaknesses in TB care, including diagnostic delays, poor adherence, comorbid conditions, and social vulnerabilities. The WHO has set ambitious global TB targets, including a 95% reduction in TB-related deaths and a 90% decline in TB incidence by 2035, compared to 2015 levels [6]. Achieving these targets requires more than reducing incidence—it demands high-quality treatment, timely completion of therapy, and a deep understanding of the factors contributing to treatment failure in all TB patients, especially those with DS-TB.

The number of TB cases in Lithuania has decreased by 33.2% (from 37.9 cases per 100,000 population in 2019 to 25.3 cases per 100,000 population in 2023). Further, in 2019–2023, new TB cases and relapses dropped by 29.7%, while the prevalence rate decreased by 31.2%. These positive trends reflect the impact of national TB control initiatives aligned with the WHO End TB Strategy, including the “National Public Health Supervision Development Programme for 2016–2023” and the “2014–2025 Action Plan for Reducing Health Inequalities.” During the 2015–2020 period, Lithuania achieved a 49% reduction in TB incidence—more than double the WHO-recommended target of 20% [7].

In terms of treatment success, Lithuania compares favorably with many EU/EEA countries. In 2021, the EU/EEA average treatment success rate for new and relapse TB cases (excluding rifampicin-resistant TB) was 71.7%, with Lithuania reporting 85.5%—one of the highest in the region, alongside Malta (99.3%) and Norway (88.7%) [8]. That same year, Lithuania achieved an 87% success rate for new TB cases (excluding MDR/RR-TB). In contrast, the EU/EEA average declined to 64.0% in 2022, indicating a widening gap between countries and underlining the importance of sustained national efforts. Although Lithuania’s specific treatment success data for 2022 was not available in the cited source, previous years’ results suggest that the country remains above the EU/EEA average [9].

Within the EU/EEA, TB incidence is relatively low compared to global rates; however, stark inter-country disparities persist. In 2023, the average notification rate in the EU/EEA was 8.6 cases per 100,000 population, slightly higher than the 2022 average of 8.0/100,000 [7]. Lithuania’s rate of 25.3 per 100,000 places it in the intermediate-burden category. However, it remains one of the highest in the EU/EEA, second only to Romania. This emphasizes the need for continued efforts to reduce incidence and further align national progress with the WHO and EU targets [7].

In 2024, the country recorded 666 TB cases (23.08 per 100,000 population), down from 723 cases in 2023 (25.3 per 100,000), reflecting a 60% decline since 2015, according to available data [10]. Despite progress, these figures still reflect a notable public health burden, especially given the persistent risk of treatment failure among patients with DS-TB.

Treatment failure in DS-TB is not only a clinical concern—it also increases the risk of prolonged infectiousness, ongoing community transmission, and the potential development of drug resistance. While much attention is given to drug-resistant TB, failure to successfully treat DS-TB can contribute to resistance amplification. Therefore, understanding the factors contributing to DS-TB treatment failure is essential for interrupting transmission and preventing more complex, costly cases of drug-resistant TB.

Our study focused on DS-TB as a critical starting point for broader TB control strategies in Lithuania. Importantly, one of the key strengths of this study is the long-term data analysis covering a 22-year period, which is rarely available in other European countries with medium TB incidence. Previous research in such settings has often lacked longitudinal perspectives on treatment outcomes. By addressing this gap, our findings contribute valuable evidence for long-term monitoring and planning.

In parallel, an analysis of drug-resistant TB cases is currently underway, and further investigations are planned. This stepwise approach enables a more comprehensive understanding of the full TB burden and informs the development of targeted interventions at both the individual and system level.

## 2. Methods

### 2.1. Study Design

Data were collected from the Programs and Tuberculosis State Information System Department (TB Registry) from 2000 to 2021, as well as from the archives of Vilnius University Hospital Santaros Klinikos. A total of 37,713 patients were initially included in the analysis, representing all registered cases of pulmonary TB. Of these, 12,703 cases were excluded due to incorrect resistance status or other outcomes. The category “incorrect resistance status” refers to cases where the TB drug susceptibility was not clearly determined, making it impossible to classify the patient as having either drug-susceptible TB or drug-resistant TB. To ensure the accuracy and reliability of our analysis, we excluded these patients from the study. Since this group included only a small number of patients, it did not have a significant impact on the overall DR-TB results. No missing value imputation was performed, and only cases with complete information for the variables of interest were used for analysis. The final analytical dataset included 18,697 patients with pulmonary DS-TB, 5761 patients with DR-TB, and 525 patients who developed DR-TB following an initial diagnosis of DS-TB.

**Figure 1:**
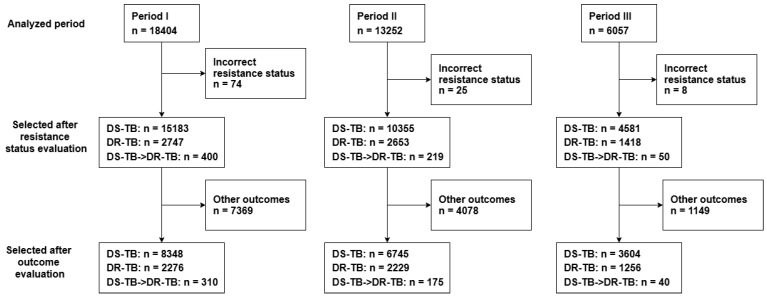
Study design flowchart. Abbreviations: DS-TB—drug-susceptible tuberculosis; DR-TB—drug-resistant tuberculosis. The category “incorrect resistance status” refers to cases where tuberculosis drug susceptibility could not be clearly determined, making it impossible to classify patients as having either drug-susceptible TB (DS-TB) or drug-resistant TB (DR-TB). To ensure the accuracy and reliability of the analysis, these patients were excluded from the study.

The study patients were divided into three periods: Period I (2000–2007), Period II (2008–2015), and Period III (2016–2021) (Figure 1) to investigate possible associations between treatment outcomes, various risk factors (such as smoking, alcohol consumption, comorbidities, etc.), and the reasons for differences in treatment outcomes across these periods. Treatment outcomes included successful treatment, defined as patients completing the treatment course and recovering, and unsuccessful treatment (patients who encountered treatment failure, died during treatment, or converted to drug-resistant tuberculosis).

### 2.2. Operational Definitions

TB cases, medication resistance, and treatment outcomes were classified by the WHO’s definitions and reporting guidelines for TB [3]. The following standardized clinical definitions were applied in Table 1.

### 2.3. Study Population

A systematic data collection form was designed to gather information from patients’ medical records. This form encompassed sociodemographic details, TB infection type, TB registration category (new patients, previously treated patients), clinical signs of TB, history of exposure to anti-tuberculosis treatment, TB treatment outcomes, and HIV status. Sputum-smear microscopy, *M. tuberculosis* cultures, and drug susceptibility testing were conducted in local laboratories, all of which underwent quality assurance by the WHO Supranational Reference Laboratory Network.

The sociodemographic and clinical characteristics of study participants are shown in Table S1. Several significant differences between the study groups were revealed. Key findings include age-related differences, particularly in the 40–50 age group, with older patients being more prevalent in Period III. There were also substantial differences in the results of microbiological tests, with Period III (98.6%) showing higher positivity rates in both culture and microscopy compared to Period I (94.9%). This was probably related to the rapid molecular tests implemented into daily clinical practice at Period III (Table S1).

Comorbidities were identified using International Classification of Diseases (ICD) codes: coronary heart disease (codes starting with I), chronic lung diseases (J44, J40, J42, J43, J47), diabetes mellitus (E10–E14), oncological conditions (codes starting with C), liver diseases (K71, K72), and kidney diseases (codes starting with N). Comorbidities also exhibit significant changes. For instance, the proportion of chronic lung diseases decreased in Period III (2.4%) compared to Period I (3.6%), whereas the rate of cardiovascular diseases increased in Period III (3.3%) compared to Period I (1.4%). Other conditions, such as diabetes mellitus (DM), kidney disease, and cancer, display higher rates in Period III (DM 2.5%, kidney disease 0.5%, and cancer 3.6%) compared to Period I (kidney disease 0.1% and cancer 0.2%). Also, significant findings include increased HIV positivity in Period III (2.3%) compared to Period I (0.2%) (Table S1).

Lifestyle factors also showed significant variation across different periods. The proportion of smokers was higher in Period III (63.9%) compared to Period I (24.6%), and the proportion of alcohol users increased in Period III (36.0%) vs. Period I (24.6%). Patients’ education levels (7.3% in Period III vs. 4.9% in Period I) and employment status (27.9% in full-time employment in Period III vs. 22.1% in Period I) also showed notable differences, with more patients working full-time in Period III (Table S1).

Social differences were apparent as well. Homelessness was less common in Period III (1.9%) than in Period I (1.3%), and a higher proportion of patients lived in rural areas in Period III (48.1%) compared to more urban dwellers in Period I (57.1%). Other significant differences include TB recurrence rates (13.1% in Period III versus 14.9% in Period I). There was also a rise in substance use history in Period III (1.4%) compared to Period I (0.3%). No significant gender-related differences were observed across the periods (*p* = 0.4641) (Table S1).

### 2.4. Statistical Analysis

All collected data were anonymized and entered into a Microsoft Excel database, https://excel.cloud.microsoft/en-us/. Statistical analysis was performed using R statistical software 4.5.1, https://www.r-project.org/. Descriptive statistics, including frequencies, proportions, and summary measures, were used to characterize the socio-demographic and clinical profiles of the study population. Categorical variables were analyzed using Pearson’s chi-square test or Fisher’s exact test, as appropriate. Continuous variables were summarized as medians with interquartile ranges (IQR).

For comparisons between two independent groups, either Student’s *t*-test or the Mann–Whitney U test was employed, depending on the normality of the data distribution. For comparisons across more than two groups, one-way analysis of variance (ANOVA) was applied; when ANOVA assumptions were not satisfied, the non-parametric Kruskal–Wallis test was used.

Univariable logistic regression analyses were conducted to examine unadjusted associations between treatment outcomes and potential explanatory variables, including socio-demographic factors, smoking status, substance use, alcohol misuse, comorbid conditions, HIV status, education level, history of TB treatment, and TB drug susceptibility. Variables with *p* < 0.05 in univariable analysis were included in multivariable logistic regression, restricted to patients with complete data. A collinearity was assessed using the variance inflation factor (VIF), and variables with high collinearity (VIF > 5) were not included in the model simultaneously. Demographical variables (age, sex) were included in the final model regardless of univariable *p*-value. Also, odds ratios (OR) with 95% confidence intervals (CI) were presented. An OR > 1 indicates increased odds of the outcome compared to the reference group, OR < 1 indicates reduced odds, and a 95% CI excluding 1 denotes statistical significance. The TB registry is managed by the same team responsible for verifying each case and entering the data, which helps ensure consistency and accuracy in handling potential confounders.

### 2.5. Ethical Statement

The study received approval from the Regional Biomedical Research Ethics Committee of Vilnius University (30 March 2023, Nr. 158200-13-652-210, Addendum No 2023-LP-28). The Regional Biomedical Research Ethics Committee of Vilnius University exempted the necessity for informed consent in retrospective studies in compliance with the Lithuanian Law on Biomedical Research Ethics. To maintain anonymity, the data were encrypted, and access was restricted to authorized personnel only to ensure patient confidentiality after anonymization.

## 3. Results

### 3.1. Socio-Demographic and Clinical Attributes of Study Participants

**Figure 2:**
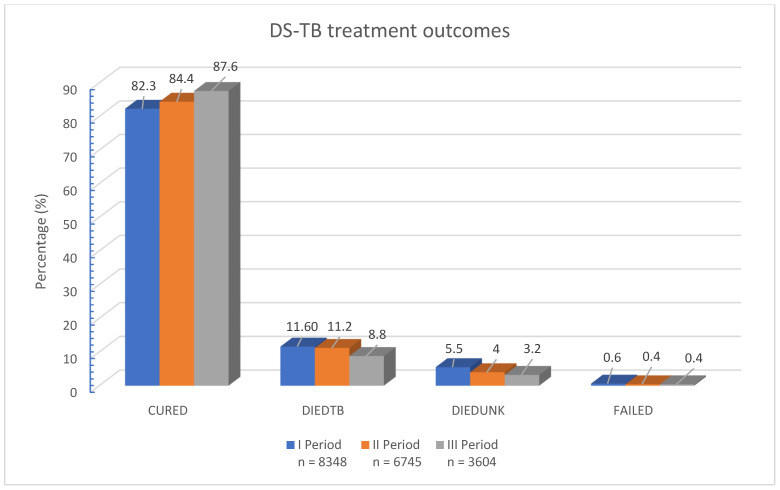
Drug-susceptible pulmonary tuberculosis treatment outcomes. Abbreviations: CURED—completed tuberculosis treatment; DIEDTB—died from tuberculosis; DIEDUNK—died from other causes; FAILED—tuberculosis treatment failure.

The treatment outcomes for pulmonary DS-TB showed a consistent and encouraging improvement over the three study periods. In Period I, out of 8348 patients, 6869 (82.3%) completed treatment. This proportion increased in Period II, with 5290 of 6745 patients (84.4%) achieving successful treatment outcomes, and further improved in Period III, where 3157 of 3604 patients (87.6%) completed treatment (Figure 2).

Conversely, the mortality rate among pulmonary DS-TB patients showed a gradual decline. In Period I, 1425 patients (17.2%) died during treatment, compared to 1028 (15.2%) in Period II and 432 (12.0%) in Period III (Figure 2).

The incidence of treatment failure remained low and stable across all periods: 54 cases (0.6%) in Period I, 27 cases (0.4%) in Period II, and 15 cases (0.4%) in Period III (Figure 2).

Among patients initially diagnosed with DS-TB, 525 were later found to have developed DR-TB. Within this subgroup, the proportion of unsuccessful treatment outcomes also declined over time: in Period I, 189 of 310 patients (61.0%) experienced unsuccessful outcomes; in Period II, 70 of 175 patients (40.0%); and in Period III, 13 of 40 patients (32.5%).

Further analysis revealed that treatment failures in DS-TB cases are significantly associated with various risk factors. These include advanced age, HIV co-infection, previous TB treatment, and socio-economic factors such as malnutrition and substance abuse. A comprehensive overview of treatment success and failure rates across the three periods is presented in Figure 2, providing a visual representation of the trends and facilitating a clearer understanding of the data.

### 3.2. A Comparative Analysis of Sociodemographic and Clinical Characteristics Between Patients with Unsuccessful TB Treatment and Treatment Success over Three Time Periods

A comparative analysis of sociodemographic and clinical characteristics was conducted between patients with unsuccessful TB treatment and treatment success in Lithuania over three time periods: 2000–2008 (Table S2), 2009–2015 (Table S3), and 2016–2021 (Table S4). Across all periods, several statistically significant differences were observed between the two outcome groups, highlighting consistent and emerging risk factors associated with treatment failure.

#### 3.2.1. Age and Gender Trends

In all three periods, patients who experienced treatment failure were significantly older than those with treatment success (*p* < 0.001). The mean age among the failure group increased slightly over time: 58.0 years (2000–2008), 59.7 years (2009–2015), and 60.6 years (2016–2021), indicating aging within this vulnerable subgroup. Male gender was consistently more prevalent among treatment failure cases across all periods, although the difference reached statistical significance only in 2000–2008 (*p* < 0.001) and 2009–2015 (*p* = 0.002) (Table S2).

#### 3.2.2. Microbiological Indicators

Culture positivity was strongly associated with treatment success across all years (100% vs. 73.0% in 2000–2008, 74.8% in 2009–2015, and 88.4% in 2016–2021; *p* < 0.001 for all). Negative culture results were observed only in the treatment failure group. Similarly, microscopy positivity was more common in the treatment success group, but the gap narrowed in 2016–2021 and was no longer statistically significant (*p* = 0.100) (Table S2).

#### 3.2.3. Comorbidities

Coronary heart disease was increasingly prevalent in the failure group across all time points (8.7% in 2016–2021 vs. 2.5% in the success group, *p* < 0.001). Chronic pulmonary disease was more common in treatment failure patients, significantly so in all periods (*p* < 0.05). Oncological diseases were strongly associated with treatment failure, particularly in the most recent period (14.1% vs. 2.1%, *p* < 0.001). Kidney disease became a significant factor only in the latest period (1.8% vs. 0.3%, *p* < 0.001). Diabetes did not show a consistent association across periods. HIV positivity was consistently higher in the treatment failure group, reaching significance in 2009–2015 (2.2% vs. 0.6%, *p* < 0.001) and showing a trend toward significance in 2016–2021 (*p* = 0.055) (Table S2).

#### 3.2.4. Social Factors

Education level was a strong determinant. A significantly greater proportion of patients who experienced treatment failure had only a primary or basic education, and a higher proportion had. In contrast, higher levels of education were more prevalent among patients who completed treatment (*p* < 0.001 for all periods). Unemployment was consistently and strongly associated with treatment failure across all periods (e.g., 92.4% in 2016–2021 vs. 69.4% in the success group, *p* < 0.001). Homelessness was more frequent among treatment failure patients, with a stable association over time (3.8% vs. 1.6% in 2016–2021, *p* = 0.001). Urban vs. rural residence did not consistently influence treatment outcomes across time (Table S2).

#### 3.2.5. Harmful Habits

Alcohol abuse was significantly more prevalent in the treatment failure group during all periods (e.g., 44.8% vs. 23.9% in 2016–2021, *p* < 0.001). Smoking was consistently more frequent in the failure group, though statistically significant only in the first and last periods. Substance use showed a significant association only in the latest period (2.5% vs. 1.2%, *p* = 0.038), potentially indicating emerging trends (Table S2).

#### 3.2.6. TB History and Relapse

New TB cases were more common among treatment success patients, while relapses or other previously treated patients were more frequent among failures (*p* < 0.01 in 2000–2015). Family TB contact was more common among patients with successful outcomes. In contrast, failure cases often reported unknown contact history, indicating potential delays in case detection or incomplete contact tracing (Table S2).

This 22-year analysis demonstrates that treatment failure in TB patients in Lithuania is consistently associated with older age, male gender, lower education, unemployment, homelessness, alcohol abuse, chronic comorbidities (especially cardiovascular, oncological, and pulmonary diseases), and relapse cases. Sociodemographic and clinical risk profiles remained relatively stable over time, though some indicators—such as oncology, kidney disease, and substance abuse—have become increasingly prominent in more recent years.

### 3.3. Drug-Susceptible Pulmonary Tuberculosis Treatment Failure Risk Factors

This study identifies and examines the most relevant statistically significant risk factors for treatment failure in patients diagnosed with pulmonary DS-TB across three distinct periods, as illustrated in Table 2. A binary logistic regression model with stepwise selection was used to evaluate the impact of clinical, demographic, behavioral, and socioeconomic variables on treatment outcomes. Odds ratios (ORs) with corresponding 95% confidence intervals (CIs) and *p*-values were reported. An OR > 1 indicates increased odds of treatment failure, while an OR < 1 suggests decreased odds. A 95% CI that does not include 1, along with a *p*-value < 0.05, was considered statistically significant.

The proportion of pulmonary DS-TB patients experiencing treatment failure decreased steadily over time from 17.7% in Period I, to 15.6% in Period II, and 12.4% in Period III. This trend highlights continuous improvements in TB diagnostics, care, and management. However, several risk factors remain significantly associated with poor treatment outcomes (Table 2).

#### 3.3.1. Age

Advanced age was consistently associated with an increased risk of treatment failure across all three periods, with ORs ranging from 1.04 to 1.05 (*p* < 0.001 in all periods; CI: 1.04–1.05). Although the effect size was modest, the association was statistically significant and likely reflects multiple age-related factors such as immunosenescence, frailty, and higher prevalence of comorbidities (Table 2).

#### 3.3.2. Sex

Male sex showed a statistically significant association with treatment failure in Periods I and II (OR = 1.59, 95% CI: 1.40–1.82, *p* < 0.001; and OR = 1.27, 95% CI: 1.09–1.48, *p* = 0.002, respectively). The association was not statistically significant in Period III (OR = 1.16, 95% CI: 0.93–1.45, *p* = 0.206) (Table 2).

#### 3.3.3. Body Mass Index

Low BMI (<18), indicating undernutrition, was a strong predictor of treatment failure in Periods II and III (OR = 11.5, 95% CI: 3.13–55.5, *p* < 0.001 in both). Data were not available for Period I (Table 2).

#### 3.3.4. Comorbidities

Comorbidities—such as coronary heart disease, chronic pulmonary disease, diabetes, cancer, kidney disease, and HIV—also played a significant role in treatment failure (Table 1). Coronary heart disease was associated with increased risk in all periods: Period I (OR = 1.54, 95% CI: 0.98–2.34, *p* = 0.049), Period II (OR = 7.83, 95% CI: 3.97–15.9, *p* < 0.001), and Period III (OR = 3.72, 95% CI: 2.48–5.50, *p* < 0.001). Chronic lung disease also increased the risk significantly: Period I (OR = 1.78, 95% CI: 1.36–2.31, *p* < 0.001), Period II (OR = 2.77, 95% CI: 1.89–3.99, *p* < 0.001), Period III (OR = 1.90, 95% CI: 1.11–3.12, *p* = 0.015). Diabetes mellitus was significant only in Period I (OR = 2.28, 95% CI: 1.56–3.29, *p* < 0.001); no significant association was found in Periods II (*p* = 0.433) or III (*p* = 0.524). Cancer was a consistent and strong risk factor: Period I (OR = 4.66, 95% CI: 1.59–13.6, *p* = 0.004), Period II (OR = 7.48, 95% CI: 4.50–12.6, *p* < 0.001), and Period III (OR = 7.80, 95% CI: 5.43–11.2, *p* < 0.001). Kidney disease was not statistically significant in Period I (*p* = 0.318), but became significant in Period II (OR = 5.41, 95% CI: 1.00–29.2, *p* = 0.039) and Period III (OR = 5.73, 95% CI: 2.18–14.6, *p* < 0.001). HIV infection was significantly associated with treatment failure in Period I (OR = 5.71, 95% CI: 2.36–14.2, *p* < 0.001) and Period II (OR = 3.60, 95% CI: 2.09–6.08, *p* < 0.001), but not in Period III (*p* = 0.057). Liver disease was not found to be a significant risk factor in any period (Table 2).

#### 3.3.5. Harmful Habits

Although smoking, excessive alcohol consumption, and substance use were identified as risk factors, their impact on treatment success was less pronounced than the factors mentioned above. Smoking increased the risk in Period I (OR = 1.29, 95% CI: 1.15–1.46, *p* < 0.001) and decreased it in Period III (OR = 0.79, 95% CI: 0.65–0.97, *p* = 0.023); it was not significant in Period II (*p* = 0.779). Excessive alcohol consumption was significantly associated with treatment failure in all periods: Period I (OR = 1.88, 95% CI: 1.62–2.17, *p* < 0.001), Period II (OR = 1.45, 95% CI: 1.23–1.71, *p* < 0.001), and Period III (OR = 2.15, 95% CI: 1.70–2.72, *p* < 0.001). Substance use was significant in Period I (OR = 2.33, 95% CI: 1.00–5.07, *p* = 0.039) and Period III (OR = 2.02, 95% CI: 0.98–3.83, *p* = 0.042); not significant in Period II (*p* = 0.687) (Table 2).

#### 3.3.6. Social Factors

Social determinants’ role in pulmonary DS-TB treatment failure (Table 1). Low education was consistently significant: Period I (OR = 2.56–7.03, 95% CI: 1.71–11.1, *p* < 0.001), Period II (OR = 1.63–5.03, 95% CI: 1.13–7.65, *p* < 0.001), and Period III (OR = 1.90–3.81, 95% CI: 1.16–7.17, *p* < 0.001). Unemployment showed a very strong and significant association across all periods: Period I (OR = 7.32, 95% CI: 5.73–9.51, *p* < 0.001), Period II (OR = 5.4, 95% CI: 4.13–7.21, *p* < 0.001), Period III (OR = 5.36, 95% CI: 3.81–7.81, *p* < 0.001). Homelessness was also a strong predictor: Period I (OR = 3.99, 95% CI: 2.77–5.72, *p* < 0.001), Period II (OR = 2.30, 95% CI: 1.61–3.23, *p* < 0.001), and Period III (OR = 2.41, 95% CI: 1.34–4.13, *p* = 0.002) (Table 2).

#### 3.3.7. TB History and Relapse

A history of previous TB treatment (i.e., relapse) significantly increased the risk of treatment failure in Period I (OR = 1.87, 95% CI: 1.62–2.15, *p* < 0.001) and Period II (OR = 1.31, 95% CI: 1.09–1.57, *p* = 0.004). No significant association was observed in Period III (*p* = 0.690) (Table 2).

#### 3.3.8. Factors Without a Significant Association

Some factors, including moderate alcohol consumption, liver disease, and rural residence, did not show statistically significant associations with treatment failure. Moderate alcohol consumption was associated with reduced odds of treatment failure across all periods: Period I (OR = 0.66, 95% CI: 0.56–0.78, *p* < 0.001), Period II (OR = 0.52, 95% CI: 0.43–0.63, *p* < 0.001), and Period III (OR = 0.67, 95% CI: 0.51–0.87, *p* = 0.003), suggesting a potential protective effect. Living in a rural area showed a statistically significant association only in Period I (OR = 1.30, 95% CI: 1.16–1.46, *p* < 0.001); not significant in Periods II (*p* = 0.815) or III (*p* = 0.303). Liver disease was not a significant factor in any period (Table 2).

This comprehensive analysis reveals a multifactorial landscape of risk for treatment failure in pulmonary DS-TB. While overall treatment outcomes improved over time, patients with advanced age, comorbidities, substance use issues, and adverse social conditions remained at elevated risk. Subgroup analyses across age, sex, comorbidity, and social factors—including both *p*-values and confidence intervals—demonstrate the importance of personalized and targeted interventions.

**Table 2:** Demographic, clinical, and laboratory characteristics of the study patients.

Risk Factor			Patients (N = 18,697)			
	Period I (N = 8348)		Period II (N = 6745)		Period III (N = 3604)	
	OR (95% CI)	*p*	OR (95% CI)	*p*	OR (95% CI)	*p*
Aged 50 and above	1.04 (1.04–1.04)	<0.001	1.05 (1.04–1.05)	<0.001	1.05 (1.04–1.05)	<0.001
Confirmed by culture	0.68 (0.60–0.76)	<0.001	0.74 (0.65–0.85)	<0.001	-	-
Low BMI (<18)	-	-	11.5 (3.13–55.5)	<0.001	11.5 (3.13–55.5)	<0.001
Coronary heart disease	1.54 (0.98–2.34)	0.049	7.83 (3.97–15.9)	<0.001	3.72 (2.48–5.50)	<0.001
Chronic lung disease	1.78 (1.36–2.31)	<0.001	2.77 (1.89–3.99)	<0.001	1.9 (1.11–3.12)	0.015
Diabetes mellitus	2.28 (1.56–3.29)	<0.001	-	0.433	-	0.524
Cancer disease	4.66 (1.59–13.6)	0.004	7.48 (4.50–12.6)	<0.001	7.8 (5.43–11.2)	<0.001
Kidney disease	-	0.318	5.41 (1.00–29.2)	0.039	5.73 (2.18–14.6)	<0.001
Liver disease	-	-	-	-	-	-
HIV	5.71 (2.36–14.2)	<0.001	3.60 (2.09–6.08)	<0.001	-	0.057
Smoking	1.29 (1.15–1.46)	<0.001	-	0.779	0.79 (0.65–0.97)	0.023
Excessive alcohol consumption	1.88 (1.62–2.17)	<0.001	1.45 (1.23–1.71)	<0.001	2.15 (1.70–2.72)	<0.001
Moderate alcohol consumption	0.66 (0.56–0.78)	<0.001	0.52 (0.43–0.63)	<0.001	0.67 (0.51–0.87)	0.003
Substance use	2.33 (1.00–5.07)	0.039	-	0.687	2.02 (0.98–3.83)	0.042
Low level of education *	2.56–7.03 (1.71–11.1)	<0.001	1.63–5.03 (1.13–7.65)	<0.001	1.90–3.81 (1.16–7.17)	<0.001
Unemployment	7.32 (5.73–9.51)	<0.001	5.4 (4.13–7.21)	<0.001	5.36 (3.81–7.81)	<0.001
Homelessness	3.99 (2.77–5.72)	<0.001	2.30 (1.61–3.23)	<0.001	2.41 (1.34–4.13)	0.002
Living in a rural area	1.30 (1.16–1.46)	<0.001	-	0.815	-	0.303
TB relapse	1.87 (1.62–2.15)	<0.001	1.31 (1.09–1.57)	0.004	-	0.690
Male sex	1.59 (1.40–1.82)	<0.001	1.27 (1.09–1.48)	0.002	1.16 (0.93–1.45)	0.206
Treatment failure, n (%)	1479 (17.7)		1055 (15.6)		447 (12.4)	

Abbreviations: OR—odds ratio (expressed unless otherwise specified); * indicates a range of odds ratios in this row; CI = Confidence Interval; BMI—body mass index; HIV—human immunodeficiency virus.

### 3.4. Risk Factors for Unsuccessful Pulmonary Drug-Susceptible Tuberculosis Treatment Outcomes: An Univariable Analysis Across Three Periods

Using univariable analysis, our study examines the significant risk factors associated with unsuccessful pulmonary TB treatment outcomes across three distinct periods. The findings highlight temporal shifts in the impact of clinical, behavioral, and social determinants on treatment outcomes. Also, odds ratios (OR) with 95% confidence intervals (CI) were presented. An OR > 1 indicates increased odds of the outcome compared to the reference group, OR < 1 indicates reduced odds, and a 95% CI excluding 1 denotes statistical significance.

In the first period, the most important factors included HIV infection (OR = 5.71; 95% CI: 2.36–14.2; *p* < 0.001), drug addiction (OR = 2.33; 95% CI: 1.00–5.07; *p* = 0.039), homelessness (OR = 3.99; 95% CI: 2.77–5.72; *p* < 0.001), and cancer (OR = 4.66; 95% CI: 1.59–13.6; *p* = 0.004). Age (OR = 1.04; 95% CI: 1.04–1.04; *p* < 0.001), male sex (OR = 1.59; 95% CI: 1.40–1.82; *p* < 0.001), chronic pulmonary diseases (OR = 1.78; 95% CI: 1.36–2.31; *p* < 0.001), diabetes mellitus (OR = 2.28; 95% CI: 1.56–3.29; *p* < 0.001), smoking (OR = 1.29; 95% CI: 1.15–1.46; *p* < 0.001), heavy alcohol consumption (OR = 1.88; 95% CI: 1.62–2.17; *p* < 0.001), and unemployment (OR = 7.32; 95% CI: 5.73–9.51; *p* < 0.001) were also identified as significant risk factors that increase the likelihood of TB treatment failure. People with lower education levels (OR = 2.56–7.03), those living in rural areas (OR = 1.30; 95% CI: 1.16–1.46; *p* < 0.001), and those with a previous history of TB recurrence (OR = 1.87; 95% CI: 1.62–2.15; *p* < 0.001) also had a higher risk, further increasing the likelihood of treatment failure. Microscopic examinations and certain social factors, such as unemployment (OR = 7.32; 95% CI: 5.73–9.51; *p* < 0.001), were associated with an increased risk of TB treatment failure (Figure S1).

In the second period, the following factors were significantly associated with TB treatment failure: age (OR = 1.05; 95% CI: 1.04–1.05; *p* < 0.001), male sex (OR = 1.27, 95% CI: 1.09–1.48; *p* = 0.002), chronic diseases (e.g., coronary heart disease (OR = 7.83; 95% CI: 3.97–15.9; *p* < 0.00), chronic lung diseases (OR = 2.77; 95% CI: 1.89–3.99; *p* < 0.001), kidney diseases (OR = 5.41; 95% CI: 1.00–29.2; *p* = 0.039), cancer (OR = 7.48; 95% CI: 4.50–12.6; *p* < 0.001). HIV infection (OR = 3.60; 95% CI: 2.09–6.08), alcohol abuse (OR = 1.45; 95% CI: 1.23–1.71; *p* < 0.001). low education (OR = 1.63–5.03), unemployment (OR = 5.40; 95% CI: 4.13–7.21; *p* < 0.001). Homelessness (OR = 2.30; 95% CI: 1.61–3.23; *p* < 0.001), a previous history of TB recurrence (OR = 1.31; 95% CI: 1.09–1.57; *p* = 0.004) are all statistically significant risk factors, significantly increasing the TB treatment failure. Conversely, diabetes mellitus, kidney diseases, smoking, drug addiction, and living in rural areas were not found to have a significant impact on TB treatment failure (Figure S2).

The study identified several factors significantly associated with TB treatment failure risk in the third period. Age increases the likelihood of developing TB by 5% per year, with a statistically significant relationship (OR = 1.05, *p* < 0.001). Male sex (OR = 1.16; 95% CI: 0.93–1.45; *p* = 0.206), coronary heart disease (OR = 3.72; 95% CI: 2.48–5.50; *p* < 0.001), cancer (OR = 7.80; 95% CI: 5.43–11.2; *p* < 0.001). Chronic lung diseases (OR = 1.90; 95% CI: 1.11–3.12; *p* = 0.015) and lower education level (OR = 1.90–3.81) significantly increase TB risk failure. For instance, individuals with cancer have nearly eight times the risk (OR = 7.80, *p* < 0.001), and those with kidney disease have over five times the risk (OR = 5.73, *p* < 0.001). Alcohol abuse (OR = 2.15; 95% CI: 1.70–2.72; *p* < 0.001) and drug addiction (OR = 2.02; 95% CI: 0.98–3.83; *p*–0.042) also notably raise the risk of TB treatment failure, while diabetes mellitus, kidney diseases, smoking, HIV, living in rural areas, a previous history of TB recurrence have less substantial or statistically insignificant impacts. Social factors such as unemployment and homelessness are essential contributors to TB treatment failure, as unemployed individuals face more than five times the risk (OR = 5.36; 95% CI: 3.81–7.81; *p* < 0.001), and homelessness double the risk (OR = 2.41; 95% CI: 1.34–4.13; *p* = 0.002) (Figure S3).

In the latest period, there were more older individuals (aged 50 and older) and fewer younger individuals compared to the first two periods. In the third period, there were more individuals with culture-confirmed tuberculosis. Patients in the third period were more likely to have coronary heart disease, diabetes, cancer, and kidney disease. These patients were better educated, consumed less alcohol, and were more likely to be employed. However, they were more likely to use drugs and were infected with HIV. Treatment effectiveness improved over time, with a decrease in treatment failures and mortality. Statistical analysis suggests these changes are not random and may have significant implications for future treatment strategies.

### 3.5. Multivariable Logistic Regression Results for Pulmonary Drug-Susceptible Tuberculosis Treatment Outcome Predictors

**Figure 3:**
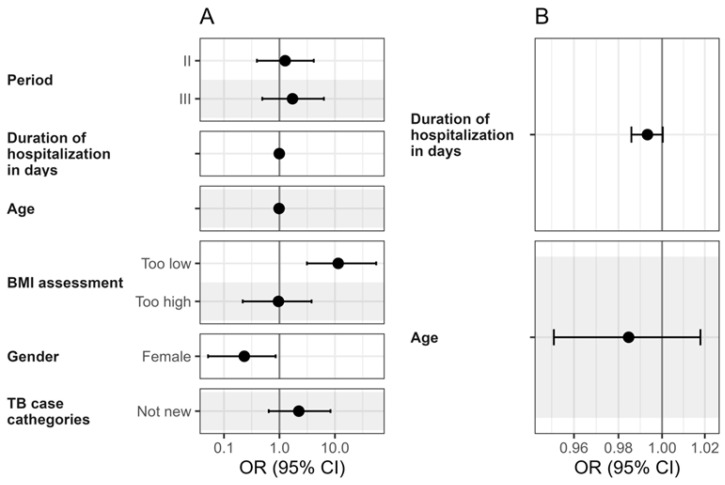
Multivariable analysis of factors associated with drug-susceptible pulmonary tuberculosis and unsuccessful treatment outcomes. (**A**): full model; (**B**): zoomed part of A for better visibility of confidence intervals. Abbreviations: OR = odds ratio; CI = confidence interval.

The results of a multivariable logistic regression analysis examining key factors associated with pulmonary TB treatment outcomes are presented in Figure 3. In this model, BMI was included as a categorical variable (<18 vs. ≥18), alongside sex and potential confounding variables such as age, period, length of hospitalization, and TB case category (new vs. previously treated). The analysis identified low BMI (<18) as a strong independent predictor of unsuccessful treatment outcomes. Patients with low BMI had an OR of 11.5 (*p* < 0.001), indicating a significantly increased risk of treatment failure.

Sex also emerged as a significant factor. Women were less likely to experience adverse outcomes than men, with an OR of 0.23 (95% CI: 0.05–0.86, *p* = 0.038) (Figure 3).

The length of hospitalization was associated with treatment outcomes, though the effect size was modest. More extended hospitalization was linked to a slightly reduced risk of treatment failure (OR = 0.99, *p* = 0.047) (Figure 3).

In contrast, age did not show a statistically significant effect on treatment outcomes (*p* > 0.05), nor did TB case newness (new vs. previously treated), although the OR for previously treated cases (OR = 2.23, *p* = 0.225) suggests a potential trend toward poorer outcomes that did not reach statistical significance (Figure 3).

Overall, the multivariable analysis confirms that low BMI is the most significant and consistent predictor of TB treatment failure in this cohort. Sex also plays a meaningful role, with women showing better outcomes. Other factors, including age, period, and prior TB history, did not significantly impact the results in this model. These findings highlight the importance of targeted interventions to address malnutrition and to consider sex-specific approaches in TB treatment strategies (Figure 3).

## 4. Discussion

Our study shows that over 22 years, the number of pulmonary DS-TB cases has steadily decreased. Correspondingly, the pulmonary DS-TB treatment failure rate decreased from 17.7% in the first period to 15.6% in the second and 12.4% in the third. These findings indicate a growing effectiveness of TB management and a reduction in treatment failures, signaling a steady improvement in the outcomes for pulmonary DS-TB.

The key findings of our study are that the leading causes of pulmonary DS-TB treatment failure are low BMI, male sex, older age, comorbidities (coronary heart disease, chronic lung disease, diabetes mellitus, oncological diseases, kidney disease, HIV), social factors (low education, lack of employment, lack of housing, smoking), and TB relapse. Other important risk factors: substance use, excessive alcohol consumption (less significant than the leading causes). These are discussed below.

Our findings further support the role of low body mass index (BMI < 18 kg/m^2^) as a key contributor to treatment failure in TB. In an observational study of 264 patients, Lawrence et al. [11] found that a low BMI at the initiation of treatment for rifampicin- or multidrug-resistant TB (MDR/RR-TB) is associated with poorer treatment outcomes. A moderate correlation was observed between low BMI and reduced likelihood of treatment success. Additionally, individuals with a BMI below 24 kg/m^2^ were found to have a 2.68-fold increased risk of developing TB, emphasizing the importance of nutritional status in disease progression and treatment response [11]. The evidence suggests that improved nutritional health, particularly increased BMI, may facilitate faster culture conversion and improve treatment outcomes [11]. Clinical trials evaluating micronutrient supplementation during TB therapy have demonstrated modest benefits, including a 2–3 kg weight gain after two months of treatment, along with potential enhancements in physical function, sputum conversion rates, and treatment completion [12]. These findings underscore the critical role of malnutrition—specifically low BMI—in influencing TB outcomes [13]. Based on this evidence, we recommend that patients with low BMI receive targeted nutritional support, emphasizing increased protein intake, to enhance treatment efficacy and improve clinical outcomes.

In our study, male gender and older age were also identified as contributing factors to treatment failure. These findings are consistent with the results reported by AlOsaimi et al. [14], who analyzed data from the National Tuberculosis Registry of Saudi Arabia to assess treatment outcomes in 427 patients with pulmonary TB. Treatment outcomes were classified as successful or unsuccessful based on clinical evaluation, radiographic findings, and sputum examination during follow-up. The study reported a treatment success rate of 88.5%. However, male patients were more likely to experience treatment failure compared to females, with an aOR of 1.3, indicating that men were 1.3 times more likely to have an unsuccessful outcome. Notably, the rate of treatment failure was higher among individuals aged 65 years and older. Our findings are in alignment with these observations. Given the increased risk of poor outcomes in these populations, directly observed therapy (DOT) is strongly recommended for male patients and older adults to promote adherence and improve treatment success rates.

Several comorbidities emerged as significant risk factors for TB treatment failure in our study. These findings align with previously published research. For example, Matulyte et al. [15] conducted a retrospective chart review in Lithuania using data from the national TB Register to examine the characteristics of individuals co-infected with TB and HIV. Lithuania remains one of the 18 high-priority TB countries in the WHO European region, and since 2015, TB has been the most prevalent AIDS-indicative disease in the EU/EEA. The study analyzed 345 TB-HIV co-infection cases among 311 individuals, including 239 new and 106 previously treated TB patients. Treatment success rates were notably low, at 61.4% for DS-TB. The corresponding treatment failure rates—38.6% for DS-TB—highlighted the challenges in managing TB-HIV co-infection in this population. Our findings further support that HIV infection is a statistically significant predictor of TB treatment failure and emphasize the need for integrated care and close monitoring of patients co-infected with TB and HIV.

Xiao et al. [16] investigated the impact of CKD on TB outcomes in 167 patients treated at tertiary centers in China. Higher mortality rates were observed among CKD patients, especially those in advanced stages or undergoing hemodialysis. Multivariate analysis identified CKD stage 4–5, age ≥ 40 years, hypoalbuminemia, and hemodialysis as independent risk factors for mortality. These findings support our observation that CKD significantly increases the risk of poor TB treatment outcomes.

The impact of diabetes mellitus (DM) on TB treatment outcomes has also been well documented. A systematic review by Huangfu et al. [17] involving over 56,000 TB-DM patients found significantly higher odds of mortality (OR 1.88), relapse (OR 1.64), and MDR-TB (OR 1.98) compared to TB patients without diabetes. Gautam et al. [18] conducted a similar analysis in South Asia, reporting a 21% pooled prevalence of DM among TB patients, and found increased risks of mortality and treatment failure (OR 1.7 for both). Nowiński et al. [19] further confirmed the detrimental impact of diabetes, reporting an OR of 1.9 for TB-related mortality in a national cohort study of over 19,000 Polish patients. In addition, a recent study by Cioboata et al. [20] emphasized the biological mechanisms linking metabolic dysfunction with TB progression and outcomes, highlighting the role of host metabolic markers such as lipid metabolism and immune modulation in TB pathogenesis. Similarly, Huangfu et al. [21] provided evidence from high TB burden settings, demonstrating how diabetes impairs immune response and contributes to worse TB outcomes, particularly in low-resource countries.

Consistent with these findings, our study demonstrated that DM is a significant risk factor for TB treatment failure, highlighting the importance of integrated disease management and the need to consider metabolic comorbidities in TB control strategies.

Cancer has also emerged as a significant comorbidity. Adam Nowiński et al. [19] found that patients with cancer had a markedly increased risk of TB-related mortality (OR 3.4) and all-cause mortality (OR 15.4). Our findings corroborate this, showing that oncological diseases significantly increase the likelihood of TB treatment failure. Such patients require enhanced clinical attention and follow-up during the TB treatment course.

Regarding pulmonary conditions, Koo et al. [22] analyzed 52 TB patients with treatment failure and found that chronic lung disease and the presence of cavitary lesions on chest radiography were associated with poor treatment outcomes. Our study supports these findings, showing that chronic pulmonary diseases are significantly linked to TB treatment failure, underscoring the need for careful evaluation and follow-up of patients with underlying respiratory illnesses.

The impact of chronic coronary diseases on TB treatment outcomes has been well established. This summary integrates findings from two complementary studies that underscore the importance of addressing comorbidities in TB care. A cross-sectional study conducted within the national TB programs of Kenya, Uganda, Zambia, and Zimbabwe [23] assessed 1063 patients (78% of those eligible) to evaluate the feasibility of routine screening for comorbidities, risk factors, and disabilities. The results showed that 72% of patients had at least one such condition, with 16% diagnosed with high blood pressure (including 124 newly identified cases). Additionally, 80% of patients with disabilities required referral beyond their initial facility, emphasizing the need for integrated, patient-centered care. In a separate retrospective cohort study, Ehud Kaliner et al. [24] compared 50 TB patients with a history of substance use to 100 matched non-users treated between 2007 and 2017. Substance users had higher rates of multidrug-resistant TB, prolonged hospitalizations, increased treatment failure, and mortality. Multivariate analysis identified chronic disease as a significant predictor of treatment failure (OR = 12.4). In line with these findings, our study further demonstrates that chronic coronary disease is a critical risk factor for TB treatment failure, reinforcing the necessity of integrated disease management to improve TB outcomes.

Based on these findings, we strongly recommend comprehensive comorbidity management, particularly for coronary heart disease, chronic pulmonary disease, cancer, DM, CKD, and HIV, as part of TB treatment protocols. Our results align with existing literature and emphasize the need for individualized and multidisciplinary approaches to reduce treatment failure rates in high-risk patient groups.

In addition to clinical factors, socioeconomic determinants were also significantly associated with poor TB outcomes in our study. These results are in line with those of Yerezhepov et al. [25], who conducted a case–control study in Kazakhstan involving 1555 participants. Their analysis identified unemployment as a significant risk factor for poor treatment outcomes (χ^2^ = 81.1, *p* < 0.001). Consistent with these findings, our study also demonstrated that unemployment significantly increases the risk of TB treatment failure.

Similarly, a retrospective study by Sánchez-Pérez et al. [26] in Mexico analyzed National Epidemiological Surveillance System (SINAVE) data to assess TB incidence and treatment success or failure determinants. The study found that individuals with at least a secondary education had a markedly higher treatment success rate (88.3%) than those with only primary education or less (24.2%). Furthermore, individuals not engaged in agricultural labor had higher treatment success rates (83.6%) than those working in agriculture (29.1%). Additional sociodemographic factors associated with poorer outcomes included lower educational levels and employment in the agricultural sector. The results of our study are consistent with the study’s findings above. Low educational levels significantly increase the risk of tuberculosis treatment failure.

Ehud Kaliner et al. [24] found that substance users experienced higher rates of treatment failure, particularly those who were homeless. Multivariate analysis identified homelessness (OR = 6.7) as a strong independent predictor of treatment failure. Consistent with this, our study also found homelessness to be significantly associated with unsuccessful treatment outcomes.

These findings highlight the critical role of social determinants in shaping TB treatment outcomes. Unemployed individuals and those experiencing homelessness often face barriers to accessing care and adhering to lengthy treatment regimens. We recommend that such vulnerable populations receive integrated psychosocial and social support services, alongside medical treatment, to enhance adherence, reduce disparities, and improve clinical outcomes.

Furthermore, our study found that tobacco smoking is significantly associated with adverse TB treatment outcomes. This finding is supported by Kaliner et al. [24], who reported that nearly 70% of TB patients who smoked experienced treatment failure. Similar associations have been consistently demonstrated in multiple studies across diverse geographic settings [27,28,29], reinforcing the evidence that smoking negatively impacts TB treatment success. Smoking remains a highly prevalent risk factor among TB patients, with approximately two-thirds identified as current or former smokers. Given its detrimental effect on treatment outcomes, we recommend that TB treatment programs incorporate targeted psychological and behavioral support interventions for patients who smoke, to facilitate smoking cessation and improve overall treatment efficacy.

Additional risk factors for TB treatment failure identified in our study included substance use and excessive alcohol consumption. However, their impact was less pronounced than comorbidities, low BMI, and sociodemographic variables. Nonetheless, alcohol use demonstrated a significant adverse effect on TB treatment outcomes. In a retrospective cohort study by Kaliner et al. [24], which compared 50 TB patients who consumed alcohol with 100 non-alcoholic TB patients from 2007 to 2017, alcohol use emerged as a strong independent predictor of treatment failure (adjusted odds ratio = 4.0). Kaplan–Meier survival analysis further revealed that alcohol-consuming patients had significantly shorter survival times (*p* = 0.03), with the average age at death notably lower among alcohol users (42.9 years) compared to non-users (77.3 years). These findings suggest that alcohol consumption increases the risk of treatment failure fourfold. Substance use was also shown to compromise treatment outcomes. A retrospective cohort study by Coutinho et al. [30] in Brazil investigated smoking and substance use among adult TB outpatients. The study followed 111 patients over six months and found a smoking cessation rate of only 26.8% (19 of 71 evaluable patients). A high loss to follow-up rate (40.5%) was observed, attributed to death (n = 4) or discontinuation of care (n = 36). Patients identified multiple barriers to cessation and treatment adherence, including anxiety and depression (47.4%), exposure to others smoking (38.5%), and concurrent drug use (19.2%). Given the detrimental effects of alcohol and substance use on TB outcomes, we recommend that affected patients receive structured psychological and addiction support. Inpatient TB treatment may be necessary for these individuals to ensure adherence and improve clinical outcomes over therapy.

The burden of TB is closely linked to a range of social and behavioral determinants, including poverty, malnutrition, harmful alcohol consumption, HIV infection, smoking, and diabetes [4]. In Lithuania, approximately 64% of individuals diagnosed with TB are unemployed, 30% report harmful alcohol use, 1.8% use narcotic substances, and 12% exhibit poor adherence to prescribed treatment regimens [8]. Harmful alcohol use and poor treatment compliance have been identified as major contributors to the emergence and transmission of DR-TB [31].

Key factors contributing to treatment discontinuation among this population include lack of motivation, insufficient access to social and psychological support, and the limited availability of integrated healthcare services. In particular, there is a notable absence of coordinated care models that integrate TB diagnosis and treatment with substance use disorder management [32]. Addressing these systemic gaps is critical to improving outcomes in vulnerable populations.

The standard treatment for pulmonary DS-TB has remained essentially unchanged for decades, comprising a six-month regimen of isoniazid, rifampicin, pyrazinamide, and ethambutol (HRZE) for the first two months, followed by isoniazid and rifampicin (HR) for the remaining four months [33]. While this regimen is highly effective when adhered to, it poses challenges such as a prolonged duration, high pill burden, and potential adverse drug reactions, all of which can hinder patient adherence and contribute to the development of drug resistance. New, shorter treatment regimens have been developed in response to these limitations. The reduced treatment duration holds promise for improving adherence and reducing the risk of treatment failure [34,35].

By implementing Lithuania’s 2014–2025 National Progress Program for Health [36] and the 2014–2023 Action Plan for Reducing Health Inequalities [37], several key improvements were achieved in tuberculosis care. These programs had a significant impact on enhancing TB diagnostics, including the introduction of the DOTS (Directly Observed Treatment, Short-course) strategy. In addition, TB treatment became fully reimbursed by the healthcare system, and social support was provided to patients, helping to improve treatment adherence and overall outcomes. Other legal acts aimed at strengthening TB epidemiological surveillance, as well as providing methodological and consultative support in TB diagnosis, treatment, and control, have contributed to a substantial reduction in TB cases in recent years.

However, despite improvements in both medical and social support systems, TB control programs in Lithuania are still not comprehensively integrated with broader social services. Important challenges—such as addressing unemployment, homelessness, and substance use—remain insufficiently resolved. For example, while addiction treatment centers do exist, their impact on TB outcomes could be greater if their potential were more effectively utilized within the national TB framework.

By analyzing treatment data across three defined periods, this study provides valuable insights into the evolving risk landscape of DS-TB in Lithuania. It highlights the need for public health policies that more fully integrate TB control efforts with social support systems to better serve high-risk populations.

### Future Directions

To sustain and enhance treatment success, future TB control strategies in Lithuania should consider implementing more targeted and comprehensive interventions. These may include:

- Integration of TB services with social care programs focused on unemployment, housing, and substance use treatment;
- Community-based adherence support and outreach for vulnerable populations;
- Targeted nutritional assistance to address malnutrition and low BMI;
- Development of coordinated care models for patients with multiple risk factors and comorbidities.

Such multifaceted, patient-centered approaches are essential to address the social determinants of TB and to further accelerate progress toward TB elimination.

Nonetheless, this study has several limitations. First, its retrospective design limited access to comprehensive clinical information for all individuals. Additionally, the availability of health and treatment data varied, with potential changes in treatment practices over the 22 years and inconsistencies in data reporting across healthcare institutions (e.g., not all facilities performed the same laboratory tests). Second, some patients were excluded due to incomplete data in the TB register. As data were entered by different physicians over time, the accuracy of recorded comorbidities may be inconsistent. In our study, there was a disproportionate representation of males (~70%) and females (~30%) in the patient population, which could have contributed to skewed data interpretation. This gender imbalance aligns with findings in previous research, which highlight that men are often more affected by TB due to a complex interplay of factors. For instance, the study by da Silva et al. [38] emphasizes sociocultural and behavioral factors influencing men’s greater vulnerability, including riskier health-seeking behavior and social roles that increase exposure to TB. Similarly, the work by V. Peer et al. [39] discusses how socio-psychological factors, such as stigma and gender norms, affect access to healthcare and treatment adherence differently between men and women. Additionally, Rickman et al. [40] further underscore disparities in healthcare access and utilization, highlighting that systemic barriers often limit women’s ability to seek timely care despite lower TB prevalence. Together, these studies indicate that differences in health-seeking behavior, socio-psychological contexts, and availability of healthcare resources between genders can lead to disproportionate disease burdens and data representation. While this introduces a degree of uncertainty, it reflects the realities of working with real-world data. Third, potential data gaps and inconsistencies—such as underreporting or variation in diagnostic practices—could have influenced the reported prevalence of comorbidities and the assessment of risk factors. These factors should be considered when interpreting the study’s findings.

## 5. Conclusions

Our study revealed significant findings that pulmonary DS-TB treatment failure is strongly linked to a range of risk factors, including individual behaviors, sociodemographic conditions, and comorbidities. Our multivariate analysis demonstrated that the presence of multiple risk factors—particularly comorbidities such as diabetes, chronic lung disease, and liver disease—significantly increased the odds of unsuccessful treatment outcomes. A clear dose–response relationship was observed, with the risk of treatment failure rising alongside the number of coexisting risk factors, as reflected in progressively higher odds ratios.

Patients with lower BMI, male gender, unemployment, homelessness, and behaviors such as smoking, alcohol consumption, and drug use were found to be at particularly high risk. Comorbidities, including cancer, coronary heart disease, HIV, and renal failure, further increased the likelihood of poor outcomes.

In light of these findings, patients presenting with multiple risk factors should be closely monitored and managed through personalized care plans. Practical interventions shown to be effective in similar settings include targeted nutritional support for undernourished individuals, housing programs for homeless patients, and integrated smoking cessation services as part of TB care. These supportive measures—when combined with medical treatment—can significantly improve treatment adherence and outcomes. Implementing such strategies within the framework of TB control programs could enhance care for high-risk populations and contribute to improved national TB outcomes.

## Figures and Tables

**Table 1:** The WHO definitions used for the classification of TB cases.

Term	Definition
Bacteriologically confirmed TB	TB case confirmed by positive smear microscopy, culture, or WHO-recommended rapid diagnostic tests (e.g., Xpert MTB/RIF).
Clinically diagnosed TB	TB case diagnosed based on clinical, radiological, or histological findings in the absence of bacteriological confirmation.
New patient	A person who has never received TB treatment or has received it for less than one month.
Relapse patient	A person previously treated for TB who was cured or completed treatment and is now diagnosed again, due to either relapse or reinfection.

Abbreviations: TB—tuberculosis.

## Data Availability

The data presented in this study are available on reasonable request from the corresponding author, the data are not publicly available due to legal reasons.

## Supplementary Materials

The following supporting information can be downloaded at https://www.mdpi.com/article/10.3390/medicina61101805/s1, Table S1: Clinical characteristics of drug-susceptible pulmonary tuberculosis cases in Lithuania, 2000–2021; Table S2: Comparison of sociodemographic and clinical characteristics between patients with tuberculosis treatment failure and treatment success in Lithuania, 2000–2008; Table S3: Comparison of sociodemographic and clinical characteristics between patients with tuberculosis treatment failure and treatment success in Lithuania, 2009–2015; Table S4: Comparison of sociodemographic and clinical characteristics between patients with tuberculosis treatment failure and treatment success in Lithuania, 2016–202; Figure S1: Univariate analysis of factors associated with drug-susceptible pulmonary tuberculosis and unsuccessful treatment outcomes in Lithuania, 2000–2007 (n = 8348); Figure S2: Univariate analysis of factors associated with drug-susceptible pulmonary tuberculosis and unsuccessful treatment outcomes in Lithuania, 2008–2015 (n = 6745); Figure S3: Univariate analysis of factors associated with drug-susceptible pulmonary tuberculosis and unsuccessful treatment outcomes in Lithuania, 2016–2021 (n = 3604).
