# Supplementary material for: Risk Factors for Treatment Failure of Drug-Susceptible Pulmonary Tuberculosis in Lithuania over 22 Years
Source: Medicina (Kaunas). 2025 Oct 8;61(10):1805. doi: 10.3390/medicina61101805 (PMC12566320; doi:10.3390/medicina61101805)
Supplement: Supplementary file 1 [file medicina-61-01805-s001.zip › medicina-3801755-supplementary.pdf]

Table S1. Clinical characteristics of drug-susceptible pulmonary tuberculosis cases in Lithuania, 2000–2021.

**Variable**	1<br>N = 7876	2<br>N = 6474	3<br>N = 3571	P
Age in years n (%)				**<0.001[1]**
[0,40]	2471 (31.4)	1950 (30.1)	1017 (28.5)	
(40,50]	1896 (24.1)	1622 (25.1)	792 (22.2)	
(50,60]	1505 (19.1)	1342 (20.7)	867 (24.3)	
(60, max]	2004 (25.4)	1560 (24.1)	895 (25.1)	
Age in years n (%)				**<0.001[1]**
[0,30]	1117 (14.2)	886 (13.7)	437 (12.2)	
(30,50]	3250 (41.3)	2686 (41.5)	1372 (38.4)	
(50, max]	3509 (44.6)	2902 (44.8)	1762 (49.3)	
Age in years. Mean ± SD	49.4 ± 17.0	49.5 ± 16.9	50.1 ± 16.4	0.075[2]
Culture results. n (%)				**<0.001[1]**
Negative	399 (5.1)	264 (4.1)	51 (1.4)	
Positive	7477 (94.9)	6210 (95.9)	3519 (98.6)	
Unknown	0	0	1	
Microscopy result. n (%)				**<0.001[1]**
N	2456 (31.2)	2181 (33.7)	1275 (35.7)	
P	5420 (68.8)	4293 (66.3)	2295 (64.3)	
Unknown	0	0	1	
Coronary heart diseases. n (%)				**<0.001[1]**
No	7767 (98.6)	6440 (99.5)	3453 (96.7)	
yes	109 (1.4)	34 (0.5)	118 (3.3)	
Chronic lung diseases. n (%)				**<0.001[1]**
No	7591 (96.4)	6349 (98.1)	3484 (97.6)	
Yes	285 (3.6)	125 (1.9)	87 (2.4)	
Diabetus mellitus. n (%)				**0.002[1]**
No	7752 (98.4)	6366 (98.3)	3482 (97.5)	
Yes	124 (1.6)	108 (1.7)	89 (2.5)	
Oncological diseases. n (%)				**<0.001[1]**
No	7864 (99.8)	6414 (99.1)	3444 (96.4)	
Yes	12 (0.2)	60 (0.9)	127 (3.6)	
Liver diseases. n (%)				0.051[3]
No	7876 (100.0)	6473 (100.0)	3569 (99.9)	
Yes	0 (0.0)	1 (0.0)	2 (0.1)	
Kidney dieases. n (%)				**<0.001[1]**
No	7866 (99.9)	6468 (99.9)	3553 (99.5)	
yes	10 (0.1)	6 (0.1)	18 (0.5)	
Education. n (%)				**<0.001[1]**
Higher education	384 (4.9)	369 (5.7)	259 (7.3)	
College / vocational	1139 (14.5)	971 (15.0)	412 (11.5)	
Secondary	2857 (36.3)	2674 (41.3)	1634 (45.8)	
Basic	2084 (26.5)	1803 (27.9)	1044 (29.3)	
Primary / no formal	1409 (17.9)	656 (10.1)	220 (6.2)	
Unknown	3	1	2	
Smoking. n (%)				**<0.001[1]**
Non-smoker / ex-smoker	3138 (39.9)	2378 (36.7)	1289 (36.1)	

Variable	1<br>N = 7876	2<br>N = 6474	3<br>N = 3571	P
Smoker	4735 (60.1)	4096 (63.3)	2280 (63.9)	
Unknown	3	0	2	
Alcohol consumption. n (%)				**<0.001[1]**
Does not drink	1936 (24.6)	1401 (21.6)	1286 (36.0)	
Drinks rarely	3253 (41.3)	2679 (41.4)	1346 (37.7)	
Heavy drinker / alcoholic	2683 (34.1)	2394 (37.0)	938 (26.3)	
Unknown	4	0	1	
Employment status. n (%)				**<0.001[1]**
Employed / irregular	1744 (22.1)	1318 (20.4)	996 (27.9)	
Unemployed	6132 (77.9)	5156 (79.6)	2575 (72.1)	
Social status. n (%)				**<0.001[1]**
Not homeless	7768 (98.7)	6318 (97.6)	3503 (98.1)	
Homeless	104 (1.3)	156 (2.4)	67 (1.9)	
Unknown	4	0	1	
TB contacts. n (%)				**<0.001[1]**
Work / friends	489 (6.2)	63 (1.0)	138 (3.9)	
Family	909 (11.5)	348 (5.4)	328 (9.2)	
Unkown	6478 (82.2)	6063 (93.7)	3105 (87.0)	
Place of residence. n (%)				**<0.001[1]**
Urban	4496 (57.1)	3426 (52.9)	1855 (51.9)	
Rural	3380 (42.9)	3048 (47.1)	1716 (48.1)	
Sex. n (%)				0.464[1]
Female	2318 (29.4)	1904 (29.4)	1013 (28.4)	
Male	5558 (70.6)	4570 (70.6)	2558 (71.6)	
TB recurrence. n (%)				**<0.001[1]**
New case	6699 (85.1)	5677 (87.7)	3102 (86.9)	
Recurrence / relapse	1177 (14.9)	797 (12.3)	469 (13.1)	
Substances user. n (%)				**<0.001[1]**
No / unknown	7855 (99.7)	6443 (99.5)	3521 (98.6)	
Yes	21 (0.3)	31 (0.5)	50 (1.4)	
HIV. n (%)				**<0.001[1]**
No / unknowm	7860 (99.8)	6427 (99.3)	3488 (97.7)	
Yes	16 (0.2)	47 (0.7)	83 (2.3)	

[1]Pearson's Chi-squared test

[2]One-way analysis of means (ANOVA)

[3]Fisher's exact test

Abbreviations: N – negative; P – positive; HIV – human immunodeficiency virus.

Table S2. Comparison of sociodemographic and clinical characteristics between patients with tuberculosis treatment failure and treatment success in Lithuania, 2000–2008.

Variable	Treatment failure<br>N = 1479	Treatment success<br>N = 6869	P-value
Age in years. Mean ± SD	58.0 ± 15.5	47.0 ± 16.4	**<0.001[1]**
Culture result. n (%)			**<0.001[2]**

Variable	Treatment failure<br>N = 1479	Treatment success<br>N = 6869	P-value
N	400 (27.0)	0 (0.0)	
P	1079 (73.0)	6869 (100.0)	
Mikroskopy results. n (%)			**<0.001[2]**
N	556 (37.6)	1991 (29.0)	
P	923 (62.4)	4878 (71.0)	
Coronary heart disease. n (%)			**0.048[2]**
No	1451 (98.1)	6784 (98.8)	
Yes	28 (1.9)	85 (1.2)	
Chronic pulmonary disease. n (%)			**<0.001[2]**
No	1400 (94.7)	6658 (96.9)	
Yes	79 (5.3)	211 (3.1)	
Diabetes. n (%)			**<0.001[2]**
No	1437 (97.2)	6782 (98.7)	
Yes	42 (2.8)	87 (1.3)	
Oncology.n (%)			**0.006[3]**
No	1472 (99.5)	6862 (99.9)	
Yes	7 (0.5)	7 (0.1)	
Liver disease.last. n (%)			>0.999[3]
No	1479 (100.0)	6869 (100.0)	
Yes	0 (0.0)	0 (0.0)	
Kidney disease.last. n (%)			0.397[3]
No	1476 (99.8)	6862 (99.9)	
Yes	3 (0.2)	7 (0.1)	
Education. n (%)			**<0.001[2]**
Higher education	24 (1.6)	370 (5.4)	
College	112 (7.6)	1104 (16.1)	
No schooling / Primary	450 (30.5)	987 (14.4)	
Basic	450 (30.5)	1760 (25.6)	
Secondary	440 (29.8)	2648 (38.6)	
Unknown	3	0	
Smoking. n (%)			**<0.001[2]**
Non-smoker / Ex-smoker	497 (33.6)	2721 (39.6)	
Smoker	980 (66.4)	4147 (60.4)	
Unknown	2	1	
Alcohol. n (%)			**<0.001[2]**
Alcoholic / Abuser	776 (52.6)	2171 (31.6)	
Non-drinker	317 (21.5)	1663 (24.2)	
Rare use	383 (25.9)	3034 (44.2)	
Unknown	3	1	
Employment status. n (%)			**<0.001[2]**
Employed (regular / irregular	66 (4.5)	1750 (25.5)	
Unemployed	1413 (95.5)	5119 (74.5)	
Social status. n (%)			**<0.001[2]**
Homeless	55 (3.7)	66 (1.0)	
Not homeless	1422 (96.3)	6801 (99.0)	
Unknown	2	2	

Variable	Treatment failure N = 1479	Treatment success N = 6869	P-value
TB contacts. n (%)			**<0.001[2]**
Work / Friedns	97 (6.6)	429 (6.2)	
Unknown	1268 (85.7)	5584 (81.3)	
Family	114 (7.7)	856 (12.5)	
HIV. n (%)			**<0.001[3]**
Negative/Unknown	1468 (99.3)	6860 (99.9)	
Positive	11 (0.7)	9 (0.1)	
Substance use. n (%)			**0.043[3]**
No/Unknown	1470 (99.4)	6851 (99.7)	
Yes	9 (0.6)	18 (0.3)	
Place of residence. n (%)			**<0.001[2]**
Rural	718 (48.5)	2888 (42.0)	
Urban	761 (51.5)	3981 (58.0)	
Gender. n (%)			**<0.001[2]**
Female	319 (21.6)	2093 (30.5)	
Male	1160 (78.4)	4776 (69.5)	
TB case category. n (%)			**<0.001[2]**
New	1146 (77.5)	5944 (86.5)	
Relapse / Return	333 (22.5)	925 (13.5)	

[1]Two Sample t-test

[2]Pearson's Chi-squared test

[3]Fisher's exact test

Abbreviations: N – negative; P – positive; HIV – human immunodeficiency virus.

Table S3. Comparison of sociodemographic and clinical characteristics between patients with tuberculosis treatment failure and treatment success in Lithuania, 2009–2015.

Variable	Treatment failure N = 1055	Treatment success N = 5690	P-value
Age in years. Mean ± SD	59.7 ± 16.0	47.3 ± 16.3	**<0.001[1]**
Culture result. n (%)			**<0.001[2]**
N	266 (25.2)	0 (0.0)	
P	789 (74.8)	5690 (100.0)	
Microscopy result. n (%)			**<0.001[2]**
N	411 (39.0)	1823 (32.0)	
P	644 (61.0)	3867 (68.0)	
Coronary heart disease. n (%)			**<0.001[2]**
No	1035 (98.1)	5676 (99.8)	
Yes	20 (1.9)	14 (0.2)	
Chronic pulmonary disease. n (%)			**<0.001[2]**
No	1012 (95.9)	5604 (98.5)	
Yes	43 (4.1)	86 (1.5)	
Diabetes. n (%)			0.433[2]
No	1035 (98.1)	5601 (98.4)	
Yes	20 (1.9)	89 (1.6)	

Variable	Treatment failure<br>N = 1055	Treatment success<br>N = 5690	P-value
Oncology. n (%)			**<0.001[2]**
No	1020 (96.7)	5664 (99.5)	
Yes	35 (3.3)	26 (0.5)	
Liver disease. n (%)			0.156[3]
No	1054 (99.9)	5690 (100.0)	
Yes	1 (0.1)	0 (0.0)	
Kidney disease. n (%)			0.053[3]
No	1052 (99.7)	5687 (99.9)	
Yes	3 (0.3)	3 (0.1)	
Education. n (%)			**<0.001[2]**
Higher education	31 (2.9)	343 (6.0)	
College	93 (8.8)	921 (16.2)	
No schooling / Primary	210 (19.9)	462 (8.1)	
Basic	362 (34.3)	1520 (26.7)	
Secondary	359 (34.0)	2443 (42.9)	
Unknown	0	1	
Smoking. n (%)			0.779[2]
Non-smoker / Ex-smoker	377 (35.7)	2059 (36.2)	
Smoker	678 (64.3)	3631 (63.8)	
Alcohol. n (%)			**<0.001[2]**
Alcoholic / Abuser	560 (53.1)	1965 (34.5)	
Non-drinker	237 (22.5)	1204 (21.2)	
Rare use	258 (24.5)	2521 (44.3)	
Employment status. n (%)			**<0.001[2]**
Employed (regular / irregular	55 (5.2)	1303 (22.9)	
Unemployed	1000 (94.8)	4387 (77.1)	
Social status. n (%)			**<0.001[2]**
Homeless	47 (4.5)	113 (2.0)	
Not homeless	1008 (95.5)	5577 (98.0)	
TB contacts. n (%)			**<0.001[2]**
Work / Friends	1 (0.1)	64 (1.1)	
Unknown	1034 (98.0)	5276 (92.7)	
Family	20 (1.9)	350 (6.2)	
HIV. n (%)			**<0.001[2]**
No / Unknown	1032 (97.8)	5655 (99.4)	
Positive	23 (2.2)	35 (0.6)	
Substance use. n (%)			0.687[2]
No / Unknown	1049 (99.4)	5663 (99.5)	
Yes	6 (0.6)	27 (0.5)	
Place of residence. n (%)			0.815[2]
Rural	504 (47.8)	2696 (47.4)	
Urban	551 (52.2)	2994 (52.6)	
Gender. n (%)			**0.002[2]**
Female	265 (25.1)	1699 (29.9)	
Male	790 (74.9)	3991 (70.1)	
TB case category. n (%)			**0.004[2]**

Variable	Treatment failure N = 1055	Treatment success N = 5690	P-value
New	891 (84.5)	4989 (87.7)	
Relapse / Return	164 (15.5)	701 (12.3)	

[1]Two Sample t-test

[2]Pearson's Chi-squared test

[3]Fisher's exact test

Abbreviations: N – negative; P – positive; HIV – human immunodeficiency virus.

Table S4. Comparison of sociodemographic and clinical characteristics between patients with tuberculosis treatment failure and treatment success in Lithuania, 2016–2021.

Variable	Treatment failure N = 447	Treatment success N = 3157	P-value
Age in years.Mean ± SD	60.6 ± 15.5	48.7 ± 16.0	**<0.001[1]**
Culture result. n (%)			**<0.001[2]**
N	51 (11.4)	0 (0.0)	
P	395 (88.4)	3157 (100.0)	
Microscopy result. n (%)			0.100[2]
N	153 (34.2)	1135 (36.0)	
P	293 (65.5)	2022 (64.0)	
Coronary heart disease. n (%)			**<0.001[3]**
No	408 (91.3)	3078 (97.5)	
Yes	39 (8.7)	79 (2.5)	
Chronic pulmonary disease. n (%)			**0.013[3]**
No	428 (95.7)	3085 (97.7)	
Yes	19 (4.3)	72 (2.3)	
Diabetes. n (%)			0.523[3]
No	434 (97.1)	3081 (97.6)	
Yes	13 (2.9)	76 (2.4)	
Oncology. n (%)			**<0.001[3]**
No	384 (85.9)	3092 (97.9)	
Yes	63 (14.1)	65 (2.1)	
Liver disease. n (%)			>0.999[2]
No	447 (100.0)	3155 (99.9)	
Yes	0 (0.0)	2 (0.1)	
Kidney disease. n (%)			**<0.001[2]**
No	439 (98.2)	3147 (99.7)	
Yes	8 (1.8)	10 (0.3)	
Education char. n (%)			**<0.001[3]**
Higher education	16 (3.6)	244 (7.7)	
College	29 (6.5)	388 (12.3)	
No schooling / Primary	44 (9.9)	176 (5.6)	
Basic	173 (38.9)	882 (27.9)	
Secondary	183 (41.1)	1467 (46.5)	
Unknown	2	0	
Smoking. n (%)			**0.023[3]**

Variable	Treatment failure N = 1055	Treatment success N = 5690	P-value
Non-smoker / ex-smoker	182 (40.8)	1114 (35.3)	
Smoker	264 (59.2)	2042 (64.7)	
Unknown	1	1	
Alcohol. n (%)			**<0.001[3]**
Alcoholic / Abuser	200 (44.8)	754 (23.9)	
Non-drinker	142 (31.8)	1149 (36.4)	
Rare use	104 (23.3)	1254 (39.7)	
Unknown	1	0	
Employment status. n (%)			**<0.001[3]**
Employd (regular/irregular)	34 (7.6)	967 (30.6)	
Unemployed	413 (92.4)	2190 (69.4)	
Social status. n (%)			**0.001[3]**
Homeless	17 (3.8)	51 (1.6)	
Not homeless	429 (96.2)	3106 (98.4)	
Unknown	1	0	
TB contacs. n (%)			**<0.001[3]**
Work / Friends	10 (2.2)	128 (4.1)	
Unknown	424 (94.9)	2714 (86.0)	
Family	13 (2.9)	315 (10.0)	
HIV. n (%)			**0.055[3]**
Unknown/ negative	431 (96.4)	3090 (97.9)	
Positive	16 (3.6)	67 (2.1)	
Substance use. n (%)			**0.038[3]**
No / Unknown	436 (97.5)	3118 (98.8)	
Yes	11 (2.5)	39 (1.2)	
Place of residence. n (%)			0.303[3]
Rural	225 (50.3)	1507 (47.7)	
Urban	222 (49.7)	1650 (52.3)	
Gender. n (%)			0.206[3]
Female	115 (25.7)	903 (28.6)	
Male	332 (74.3)	2254 (71.4)	
TB case category. n (%)			0.689[3]
New	382 (85.5)	2720 (86.2)	
Relapse / Return	65 (14.5)	437 (13.8)	

[1]Two Sample t-test

[2]Fisher's exact test

[3]Pearson's Chi-squared test

Abbreviations: N – negative; P – positive; HIV – human immunodeficiency virus.

Figure S1. Univariate analysis of factors associated with drug-susceptible pulmonary tuberculosis and unsuccessful treatment outcomes in Lithuania 2000-2007 (n = 8348).

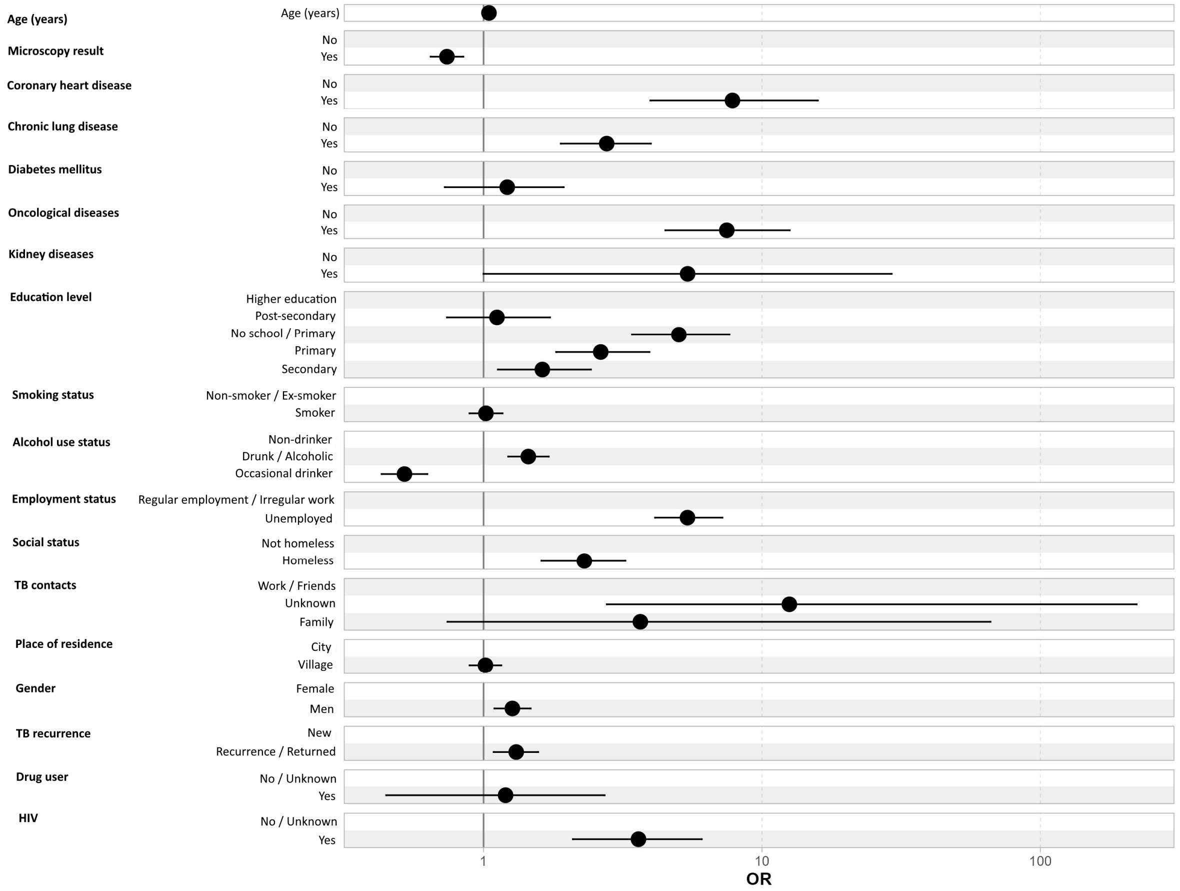

Abbreviations: OR = odds ratio; CI = confidence interval; HIV – human immunodeficiency virus.

Figure S2. Univariate analysis of factors associated with drug-susceptible pulmonary tuberculosis and unsuccessful treatment outcomes in Lithuania 2008-2015 (n = 6745).

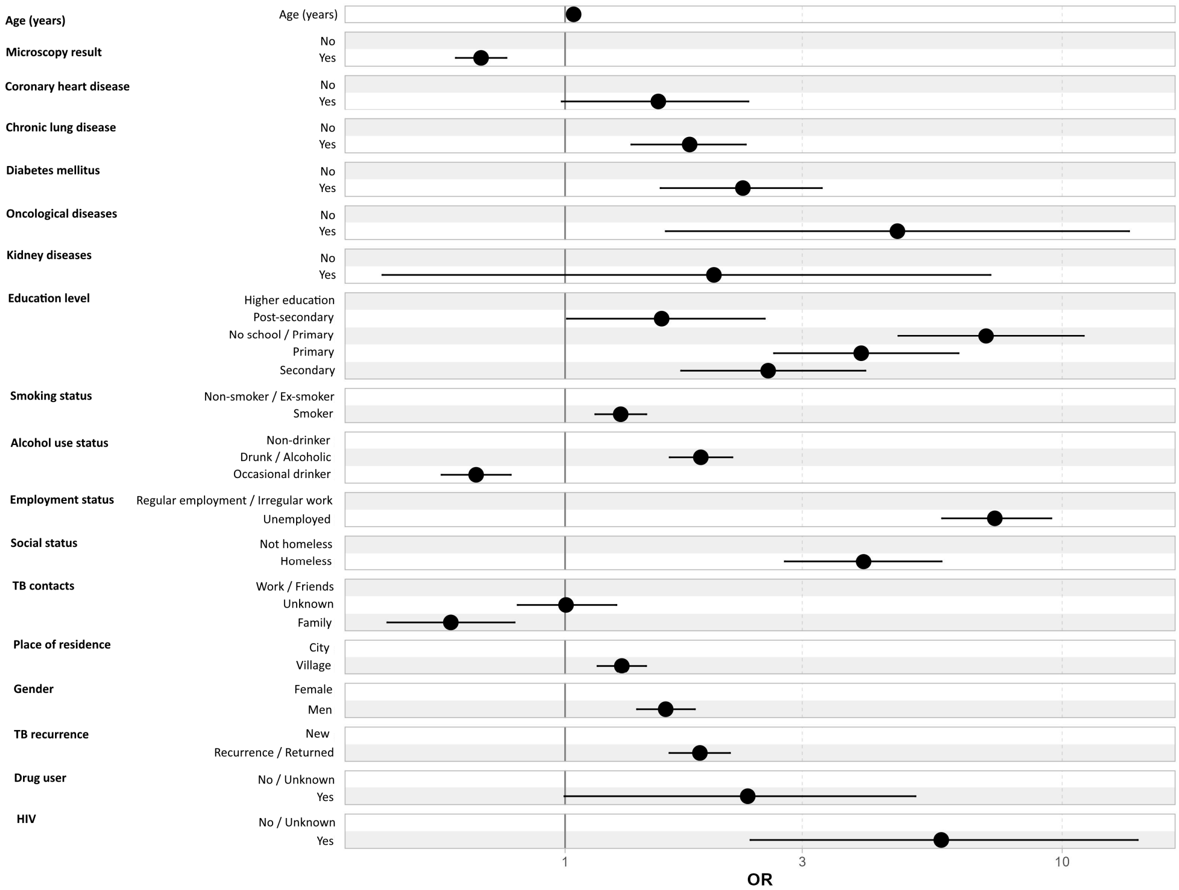

OR = odds ratio; CI = confidence interval.

Figure S3. Univariate analysis of factors associated with drug-susceptible pulmonary tuberculosis and unsuccessful treatment outcomes in Lithuania, 2016-2021 (n = 3604).

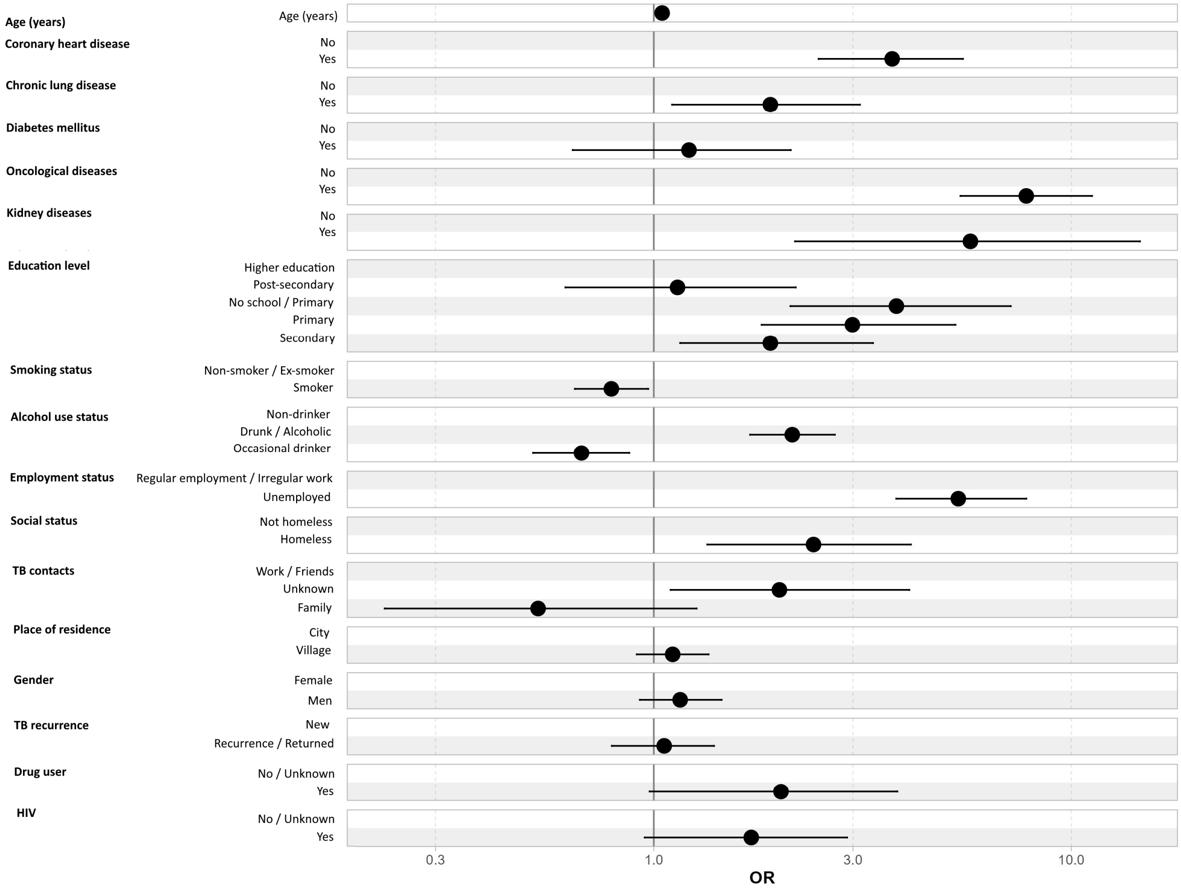

OR = odds ratio; CI = confidence interval.
